# Probiotics, prebiotics and amelioration of diseases

**DOI:** 10.1186/s12929-018-0493-6

**Published:** 2019-01-04

**Authors:** Yu-Ling Tsai, Tzu-Lung Lin, Chih-Jung Chang, Tsung-Ru Wu, Wei-Fan Lai, Chia-Chen Lu, Hsin-Chih Lai

**Affiliations:** 1 Department of Medical Biotechnology and Laboratory Science, College of Medicine, Chang Gung University, Gueishan, Taoyuan, 33302 Taiwan; 2 Microbiota Research Center and Emerging Viral Infections Research Center, Chang Gung University, Gueishan, Taoyuan, 33302 Taiwan; 3 Chang Gung Immunology Consortium, Linkou Chang Gung Memorial Hospital, Gueishan, Taoyuan, 33305 Taiwan; 4 Department of Laboratory Medicine, Linkou Chang Gung Memorial Hospital, Gueishan, Taoyuan, 33305 Taiwan; 5 Research Center for Chinese Herbal Medicine, College of Human Ecology, Chang Gung University of Science and Technology, Gueishan, Taoyuan, 33303 Taiwan; 6 Research Center for Food and Cosmetic Safety, College of Human Ecology, Chang Gung University of Science and Technology, Gueishan, Taoyuan, 33303 Taiwan; 7 Department of Medicine, College of Medicine, Chang Gung University, Gueishan, Taoyuan, 33302 Taiwan; 8 Institute of Biomedical Sciences, Academia Sinica, Taipei, 115 Taiwan; 9 Department of Respiratory Therapy, Fu Jen Catholic University, Xinzhuang, New Taipei City, 24205 Taiwan

**Keywords:** Prebiotics, Probiotics, Leaky gut, Inflammation

## Abstract

Dysbiosis of gut microbiota is closely related to occurrence of many important chronic inflammations-related diseases. So far the traditionally prescribed prebiotics and probiotics do not show significant impact on amelioration of these diseases in general. Thus the development of next generation prebiotics and probiotics designed to target specific diseases is urgently needed. In this review, we first make a brief introduction on current understandings of normal gut microbiota, microbiome, and their roles in homeostasis of mucosal immunity and gut integrity. Then, under the situation of microbiota dysbiosis, development of chronic inflammations in the intestine occurs, leading to leaky gut situation and systematic chronic inflammation in the host. These subsequently resulted in development of many important diseases such as obesity, type 2 diabetes mellitus, liver inflammations, and other diseases such as colorectal cancer (CRC), obesity-induced chronic kidney disease (CKD), the compromised lung immunity, and some on brain/neuro disorders. The strategy used to optimally implant the effective prebiotics, probiotics and the derived postbiotics for amelioration of the diseases is presented. While the effectiveness of these agents seems promising, additional studies are needed to establish recommendations for most clinical settings.

## Introduction

### Gut microbiota and microbiome

The luminal surface of the intestines contains billions of live bacteria whose total number is expected to be up to 10^14^ in colon, that is 1–100 times higher than the number of cells in an adult person [1, 2]. These bacteria form a populational density gradient, ranging from a lower density at about 10^2^/ml in the stomach, to about 10^11^/ml located in the colon. Based on the results of targeted 16S rRNA gene sequencing, there are currently 52 recognized bacterial phyla reported, with approximately five to seven phyla known to reside in the mammalian gastrointestinal tracts [3]. Among these, 4 major phyla Firmicutes, Bacteroidetes, Actinobacteria, and Proteobacteria dominate and occupy up to 97% of the total bacteria [4]. A complete and balanced bacterial ecosystem forms due to optimal interactions among the different bacterial phyla [5].

Besides the targeted 16S rRNA gene sequencing, use of the shotgun sequencing strategy provides more detailed information. All DNAs existing in the gut microbiota are sequenced. Subsequently, the open reading frames (ORFs) or genes are annotated and their functions are predicted through Gene Ontology (GO) or Kyoto Encyclopedia of Genes and Genomes (KEGG) bioinformatics resources. These may link their DNA sequences to potential biochemical metabolic pathways and functions highlighted in these bacteria [6–8]. In contrast to the term “Microbiota” that are basically phylogenetically analyzed by targeted 16S rRNA gene (mostly V3-V4 domain) sequencing, results obtained from the shotgun sequencing approach (the metagenomics approach) are named as “Microbiome”. Through metagenomics analysis, the total number of genes of the microbiota, which is predicted to be ca. 3.3 million is far more than the 25,000 from humans [9]. Thus the messages obtained from microbiome provide much more information than those from the microbiota. On top of the gut microbiome, the gastrointestinal tracts are also colonized by fungi and virus to form the gut mycobiome and the gut virome, respectively [10].

Along the life span of the humans, microbiota colonize the intestines from birth, and start to stabilize in the early first years [11]. In healthy adults, microbiota show higher complexity and diversity. By contrast, microbiota reduces diversity at elder stage [12]. There are many environmental factors influencing the microbiota composition, the most important ones being diet, way of delivery, drugs (antibiotics) usage and ageing [13].

### Gut microbiota normobiosis, dysbiosis and systemic inflammations

Gut microbiota play important roles in maintaining intestinal homeostasis, including metabolism of nutrients, synthesis of vitamin K and B12, metabolism of xenobiotics, and normal commensal bacteria prevent pathobiotic invasion and maintain barrier functions [14].

The composition of intestinal microbiota changes dynamically from birth to adulthood. Among the different phyla, Proteobacteria proliferate as a dominant phylum in newborn mice. Its number is subsequently suppressed in normal adult microbiota. The B cells and Proteobacteria-specific IgA plays an important role in the regulation of microbiota maturation and maintenance of the relative bacterial number [15]. As human beings become ageing, the percentage of Proteobacteria also increase [3]. The phylum Proteobacteria contains alpha-, beta-, gamma-, delta-, epsilon-, and zeta-proteobacteria classes. Many common human pathogens, for example, the pathogens Escherichia, Shigella, Salmonella, and Yersinia belong to the gamma-Proteobacteria [16]. On the other hand, many important gut commensals responsible for enhancing the intestinal immunity also belong to this phylum [17, 18].

Alterations in the microbiota, or the term dysbiosis, are found to be closely related to systematic inflammations and the metabolic syndromes. Among these, in adults the Proteobacteria are frequently identified to increase in many chronic inflammations-related diseases such as diabetes [19, 20], nonalcoholic fatty liver disease (NAFLD) and nonalcoholic steatohepatitis (NASH) [21], mental behaviors [22], children’s dietary behaviors [23], cardiovascular diseases [24], and colitis [25]. For example, in contrast to Firmicutes and Bacteroidetes, in liver inflammations and the prediabetes, a significant increase in the members of Enterobacteriaceae belonging to gamma-Proteobacteria was observed [26, 27].

Due to the aberrant diet habitats which are among the many causative environmental factors that lead to the situation of intestinal dysbiosis such as the over-growth of the Proteobacteria, and/or reduced Bacteroidetes, a compromised host ability to maintain a balanced gut microbial community is observed [28]. These are closely related to defective resistance of the gut commensals to colonization by enteropathogens [3]. Based on the current evidences gathered, abnormal expansion of Proteobacteria may lead to energy disequilibrium among the different bacterial species and suppression of the growth of other bacterial species. The proliferation of some bacterial species belonging to Proteobacteria may cause the development of diseases. For example, a single *Enterobacter spp*. only was reported to play a causative role in metabolic disorder. The *Enterobacter cloacae* B29 isolated from the obese human faeces, can induce obesity and insulin resistance in germ-free mice model at a monocolonization manner [29].

The change of relative abundances between different phyla may result in development of chronic inflammation. Among these, the increased Proteobacteria number may enhance chronic and systemic inflammations, leading to increased permeability of the intestine (leaky gut) and systematic inflammations in host [3]. Thus an increased prevalence of Proteobacteria may be a potential diagnostic signature of dysbiosis and the risks of disease. How to maintain the balance between these bacterial phyla to achieve immune balance is an essential issue. Basically there are many bacterial derived components that are involved in immune modulation. Among these, the increased lipopolysaccharides (LPS) derived from Proteobacteria may induce enhanced inflammations, and innate and adaptive immunity [3]. By contrast, LPS produced from Bacteroidetes generally show non-stimulatingeffects on immune cells, and may even present antagonistic effects on the LPS derived from Proteobacteria [30–32]. Such close interactions between the two bacterial phyla participate in homeostasis of the ecosystem, and are essential for the maintenance of optimal immunity, intestinal integrity and the host health.

### Traditional probiotics

Traditionally, the fermented dairy products such as the sour milk are known to show the effects of amelioration of gastroenteritis, and even the longevity [33]. Subsequently, the underlying effect and mechanism are identified to be closely related to the existence of bacteria such as lactobacilli whose fermentation products can inhibit the toxins produced by intestinal pathogens, and promote the health of cells in the host [34]. Gradually, the bacterium *Lactobacillus acidophilus* together other species and strains were shown to colonize on the surface of the human bowel, showing close interaction with the intestinal epithelial cells [35]. These issues lead to development of health-promotion bacteria called probiotics.

The initial definition of probiotics was proposed as early as in 1965 [36]. Subsequently, the WHO define that “probiotic” refers to live microorganisms that show beneficial effects on the health of the host [37]. According to the descriptions from International Scientific Association for Probiotics and Prebiotics (ISAPP), the spectrum of products that can be classified as probiotics comprise not only beneficial bacteria, but also others. These include drugs and enteral feedings for amelioration of diseases, food supplements for promotion of the benefits of health, infant formula such as the milk powders, and even the animal feedings [38].

The current definition of a probiotic indicates specific bacterial strain(s) that can effectively promote the health of humans [39]. The underlying mechanisms on how and why the bacterial strain(s) work to achieve such effects have been under intensive study [40]. Generally speaking, it is not necessary that probiotics colonize the target organ such as the intestine. However, at least certain amount of live bacteria have to reach the colon where they can affect the local intestinal ecology, physiology and metabolisms [41]. By definition, probiotics should be safe in animal, resistant to acidity and bile acids, and able to adhere and colonize in the intestine [42].

Traditionally, there are many different species of probiotics widely used. The *Saccharomyces cerevisiae* (boulardii) is the most widely used yeast strain. Other bacterial probiotics mainly comprise of *Lactobacillus species* and *Bifidobacterium species*. These include *L. rhamnosus*, *L. plantarum*, *L. sporogens*, *L. reuteri*, *L. casei*, *L. bulgaricus*, *L. delbrueckii*, *L. salivarius*, *L. johnsonii*, and *L. acidophilus*…etc. On top of these, *B. bifidum*, *B. bifidus*, *B. lactis*, *B. longum*, *B. breve (Yakult)*, and *B. infantis* are also commonly used. Other probiotics commercially available include *Streptococcus thermophilus*, *Streptococcus acidophilus*, *Lactococcus lactis*, *Enterococcus* SF68, and *Escherichia coli* Nissle 1917 (serotype O6:K5:H1) [37]. The functions of these probiotics vary significantly within the same species, mostly up to and dependent on some specific strain. Thus in evaluating the functions of the probiotics, it is essential to characterize the functions of each probiotic to the specific strain. So far the functions and effects of these probiotics in the prevention or amelioration of diseases, or in the combinational immuno-therapy basically remain controversial and need further and continuous validation. On the other hand, it is urgently needed that next generation probiotics be screened and isolated by next generation sequencing and bioinformatics platforms. These beneficial bacteria will aim for amelioration of specific and targeted diseases.

### Prebiotics and short chain fatty acids

In the 1980s, it was postulated that some components of the diet could promote the growth of certain bacterial strains present in the intestine, which are closely associated with benefits for host health [43]. Subsequently, the term “prebiotic” was generally accepted to selectively refer to food ingredients that are non-digestible and show beneficial effects on the host by stimulating the growth and/or activity of probiotics in the colon after fermentation [44]. Under this definition, there are many different kinds of food ingredients reckoned as the prebiotics. Among these, many dietary fibers which are composed of carbohydrates (polymers of mono-sugars) are most emphasized and highlighted as prebiotics. Dietary fibers basically resist the hydrolysis by human digestive enzymes in the small intestine; however, they can be fermented by colonic microbiota bacteria. Many different kinds of carbohydrates belong to dietary fibers. These include resistant starch (starch and starch degradation products), non-starch polysaccharides (celluloses, hemicelluloses, pectins, gums, and mucilages), inulin, and oligosaccharides such as fructooligosaccharides (FOS, a subgroup of inulin with the degree of polymerization (DP) ≤10), galactooligosaccharides(GOS, DP 2–8), and xylooligosaccharides (XOS, DP 2–10) [45].

Among the fermentative products of prebiotics produced from the microbiota, short chain fatty acids (SCFAs) are studied most intensively, though they may not be the only biologically active products derived from microbiota fermentation. SCFAs are mainly composed of acetate, propionate and butyrate, and many other metabolites and gases are produced after fermentation of prebiotics by microbiota bacteria [46]. SCFAs can act as energy sources absorbed through colonic mucosa [47]. Among these, acetate is mainly metabolized in muscle, kidneys, heart, and brain. Propionate undergoes metabolism in the liver and is a neoglucogenic substrate that may inhibit cholesterol synthesis and regulate lipogenesis in adipose tissue. By contrast, butyrate is mainly metabolized by the coloniccommensal bacteria, where it acts as a preferential substrate and regulates cell growth and differentiation by different mechanisms [48].

Besides the energy source, SCFAs also presented many important physiological functions, including maintaining the luminal pH, inhibiting the growth of pathogens, influencing the bowel motility, and reducing colon cancer by stimulating cancer cells apoptosis [49]. Besides, SCFAs also act as signaling molecules reducing production of proinflammatory cytokines and increasing the population of regulatory T (Treg) cells in the large intestine, through G-protein coupled receptors (GPCRs) [50]. Due to the conditions that different prebiotics produce differential amount and composition of SCFAs and gas after microbiota fermentation, for prevention or treatment for some specific inflammatory diseases, different prebiotic fibers have to be preferentially selected for administration based on their metabolic situations in the colon.

### Synbiotics

To improve the therapeutic efficacy, the “synbioitcs” are sometimes used. Synbiotics refer to food ingredients or dietary supplements composed of both probiotics and prebiotics in a form of synergism [51]. The function of synbiotics can be either complementary or synergistic. Being complementary indicates each component within the symbiotic is independently chosen for its potential health-promotion effect on host health. For example, the combination of FOS with *L. casei* in which functions from both reagents are complementary. On the other hand, being synergistic means the chosen prebiotic component is to support the activity of the specific probiotic. For example, FOS together with Bifidobacterium. More studies are needed to evaluate the optimal composition and efficacy of synbiotics, and the most optimal combination is known as “optibiotics” [52]. There are already some synbiotics used in clinical practice. These include OAT fiber/*L. plantarum*, and FOS/*L. sporogens* [51].

### Mechanisms of probiotics and prebiotics administration

The consensus of the ISAPP describes the potential underlying mechanisms of health-promotion effects from prebiotics and probiotics. These range from conserved to very unique mechanisms. General ameliorative effects include maintaining intestinal homeostasis and integrity, competitive exclusion to colonization from many other pathobionts, production of SCFAs and vitamins, metabolism of primary to secondary bile salts, regulation of gastrointestinal transit, increasing enterocyte regeneration from activation of stem cells, providing enzymes digestion activities for degradation of undigested fibers, and neutralization of carcinogens or xenobiotics….etc. [53]. These factors coming together result in enhanced integrity of the intestine and thus reduce the phenomenon of leaky gut. As the maintenance of the optimal intestinal immunity is essential, in the intestinal ecosystem, there should be neither too much inflammation nor compromised immunity in the local intestinal environment. The optimal immunity balance is achieved by maintaining the relative bacterial numbers among Bacteroidetes, Firmicutes, Proteobacteria and Actinobacteria…etc. [54]. Thus one of the main effects of administration of prebiotics and probiotics is to achieve the homeostasis of the bacterial numbers among thesephyla [55]. Based on this assumption, treatment of the prebiotics and probiotics may not just revert the imbalanced microbiota back to the same composition of the healthy subjects. Effects did present that for effective treatment from some prebiotics, probiotics or synbiotics, the compositions of microbiota are shifted towards more balanced structure [51]. This may ameliorate not only the imbalanced bacterial community, but also the aberrant blood metabolomics or cellular transcriptomics pattern of the host tissues [56].

There are also more specific mechanisms corresponding to the function of each different strain. These included modulation of neurological and brain behavior effects [57], immune-enhancing or inhibitory effects, endocrine-modulation effects, bioactive substances production, and prevention and amelioration of acute diarrhea, colitis and antibiotics associated diarrhea (AAD). Though not totally understood, it seems to adjust the intestinal back to homeostasis plays a most important role.

### Controversial effects of prebiotics and probiotics in amelioration of diseases

The effects of current prebiotics/probiotics/synbiotics on amelioration of diseases such as AAD, inflammtory bowel disease (IBD), CRC, necrotizing enterocolitis (NEC) NAFLD, encephalopathy, and ventilator-associated pneumonia (VAP) in intensive care units (ICU)…etc. remain controversial. Study results obtained are very heterogeneous and not consistent.

Among these diseases, the AAD is a very serious global clinical issue and is closely related to the *Clostridium difficile* infection after antibiotics treatment that induced gut microbiota dysbiosis [58]. Though the use of probiotics may somewhat restore intestinal microflora, the current best strategy for treatment of AAD is still through translocation of faecal microbiota (fecal microbiota transplantation, FMT) from healthy donors to the patients [59]. There are already more than 10,000 FMT cases occurring worldwide, and the number is rapidly increasing [60]. Results obtained are very positive [60]. The national clinical regulations on FMT are currently formulated and approved in many countries and it is expected that soon patients suffered from AAD are to be benefited from FMT. For some other clinical applications of probiotics and prebiotics, effects on reducing the syndromes of autism spectrum diseases (ASD) and also on the efficacy of cancer immune checkpoints therapies (ICI) [61] started to show promising results [62]. Even so, more detailed and independent basic and clinical studies are warranted.

Many other potentially deleterious effects from administration of prebiotics and probiotics were also reported. These mostly applied to patients under serious disease situations. In patients with multiorgan failure, the use of probiotics was shown to increase bacterial translocation, due to serious immunocompromised situation [63]. Furthermore, it has been indicated that the jejunal administration of probiotics with prebiotic fiber (synbiotic) in severely ill patients may possibly have negative effects on intestinal perfusion, promoting multiorgan failure, bowel necrosis, and even death [64]. Thus for the moment, it is suggested not to infuse probiotics using the jejunal administration route in critically ill patients as a standard clinical practice. More well designed and randomized studies have to be performed for detailed evaluation.

Another important issue to be mentioned is that data obtained from one study through use of the same species of probiotics or synbiotics cannot be directly extrapolated into other study. For example, *S. cerevisiae* boulardii is a widely used and studied probiotic; however, it does not show the significant activities of decreasing the risk of AAD-associated *C. difficile* infection in older patients [65]. Furthermore, effects from use of some other probiotics such as Lactobacillus, Bifidobacterium, Streptococcus, Enterococcus, and Bacillus, alone or in combination, also reported not to be effective for the elder patients suffering from AAD. A significant heterogeneity and controversy were also observed in other studies [37].

The effects of many commercial combinations of prebiotics and probiotics are also evaluated in the aspect of diarrhea. For example, *L. acidophilus*/*L. bulgaricus* 3 g/day, VSL#3 (9 × 10^11^ CFU/day), *S. boulardii* (2 g/day), *L. rhamnosus* GG (2 × 10^10^ CFU/day)/inulin 560 mg/day, Ergyphilus (2 × 10^10^ CFU/day), *L. paracasei*/*B. longum*/FOS/inulin/acacia gum, *B. breve* 1 × 10^8^/*L. casei* Shirota 1 × 10^8^/GOS 15 g, and a mixture of bifidobacteria with enteral nutrition with mixed fibers and other immunonutrients [51]. However, their effects on benefits in terms of diarrhea reduction are still not impressive. More well designed researches have to be performed to validate the effects of these treatments.

NEC is the most common serious gastrointestinal disease in preterm infants and causes the death in extremely preterm infants from 2 weeks to 2 months of age [66]. Several studies have reported the early dysbiosis with an overgrowth of intestinal *Gammaproteobacteria* in many preterm infants [67, 68]. Previous studies have shown that *Bifidobacteria species*. Are enhanced by human milk oligosaccharides in breast-fed term infants [69, 70]. By contrast, these bacteria are less common in premature infants and even less abundant in preterm infants who go on to develop NEC compared to controls [69]. Further studies utilizing *Bifidobacterium species*, *Lactobacillus species* or a combination of the two bacteria showed a strong treatment effect in reduction of NEC by cumulative meta-analysis [71, 72]. On top of these, the effects of many commercial probiotic products such as BioGaia, Culturelle…etc. that contain *Lactobacillus reuteri* and *Lactobacillus rhamnosus* GG (LGG) are also used to reduce NEC [73]. Even so, the group at greatest risk of NEC, especially those with a birthweight of < 1000 g, is relatively underrepresented in these probiotic treatment. So far we do not have adequate evidences of either efficacy or safety to recommend universal prophylactic administration of probiotics to premature infants. Hence, the effect of routine probiotics administration is controversial.

IBD classically comprising two distinct subtypes, ulcerative colitis (UC) and Crohn’s disease (CD), is characterized by chronic and relapsing inflammatory diseases of the intestines. UC by definition is continuous inflammation starting in the rectum and restricted to the colon while CD inflammation can occur anywhere in the gastrointestinal tract [74]. The microbiota composition in patients with IBD is reported to be different from that of normal individuals [75]. There are some Clostridium species producing short-chain fatty acids such as butyrate which can decrease inflammation via induction of regulatory T cells [75]. Besides, *E. coli* Nissle 1917 and the combination probiotic cocktail VSL#3 have been found to be most beneficial for UC prevention and treatment. Synbiotic, *Bifidobacterium bereve* combining with galacto-oligosaccharide, and *Bifidobacterium longum* mixing with inulinoligofructose (synergy 1) also ameliorate UC [76, 77]. Even though there are many studies, the magnitude of the effect of probiotics needs further validation.

The current studies indicate a different composition of gut microbiota between the healthy controls and many chronic inflammation-related diseases. These include obesity, diabetes, NAFLD and cardiovascular and renal diseases [78]. Changes in the composition and activity of gut microbiota after the administration of nutrients with prebiotics or probiotics may systematically change gene expression pattern (transcriptomics) and metabolism (metabolomics) of many organs in host. Organs affected many include adipose tissues, muscle, liver, pancreas, brains/neuros, lung, heart and vessels and even physiologically the modulation of satiety [79]. Administration of some prebiotics and probiotics may ameliorate the metabolic changes associated with obesity and diabetes such as insulin resistance, hyperglycemia, inflammation, dyslipidemia or NAFLD in animals [80] However, these results have to be further confirmed in humans in well-designed, controlled clinical studies. For example, the administration of probiotics (many were conducted with different strains of *Lactobacillus spp.*) may contribute to modest improvement in blood glucose control [81]. Similarly, some other reports also show that the use of prebiotics (such as GOS, FOS, inulin… etc.), probiotics, and synbiotics is associated with slight improvements in lipid control [82]. Many effects reported are still poorly relevant for clinical practice [83].

The underlying reasons that may cause heterogeneity of study results may include different populations such as adults or children, the different types and duration of antibiotics administered, the different ingredients of probiotic preparation, the different dosages used and time for each prescription, different specific strains tested, and the differential contents of the nutritional formula [84]. More evidence-based recommendations are urgently needed for administration of patients in urgent need.

### Safety of probiotics in clinical practice

The safety issue on prescription of probiotics is most important, due to the trend of rapid increase in the use of probiotics in recent years under different clinical circumstances. This is because different strains of probiotics may potentially have different safety characteristics. This issue is becoming more and more important as there may be novel probiotics to be developed soon ion the near future.

So far, few situations of bacteremia, sepsis, or endocarditis are reported to be caused by lactobacilli *L. rhamnosus* GG or *L. casei* [85]. Infections by bifidobacteria are rare in the literature. However, bacteremia, sepsis, and cholangitis induced by Bacillus subtilis have been reported [86]. On the other hand, fungal sepsis caused by S. boulardii has also been reported [55]. Basically, the risk of infection from the administration of probiotics is low and is similar to that of infection by commensal bacterial strains. Generally speaking, more benefits are observed in contrast to the risks after probiotics treatment.

Even though the probiotics generally show safety; however, for some selected groups of patients, especially for some immuno-suppressed patients, care has to be taken in use of probiotics. A number of factors predisposing to sepsis induced by administration of probiotics is proposed. Special care has to be taken for patients of severe immuno-deficiency, malnutrition or suffering from cancer [86]. For some patients who are under special treatment regime, caution also has to be take. For example, patients who have to uptake probiotics via jejunostomy, or shows symptoms of incompetent intestinal epithelial barrier (severe diarrhea) or concomitant administration of wide spectrum antibiotics [86]. All the strategies highlighted are to reduce the risks of sepsis caused by probiotics and infections by other potential pathogenic bacteria, or other diseases such as necrotizing enterocolitis in newborns [86].

## Conclusions

The use of probiotics, prebiotics, and synbiotics have been emerging as a promising therapy strategy which is generally safe in different clinical settings. While their efficacy for the prevention of diseases such as AAD, the reduction of the incidence of NEC in preterm newborns, and the prevention and treatment of UC appears to be effective, their effects are mostly marginal. More specific and disease-oriented, next generation probiotics are urgently needed. Further researches are needed before any final recommendations can be achieved.
